# The dual future of OPM-MEG: Clinical translation and naturalistic neuroscience

**DOI:** 10.1162/IMAG.a.1436

**Published:** 2026-10-29

**Authors:** Lang Qin, Miao Cao, Zhen Yuan, Bingjiang Lyu, Jia-Hong Gao

**Affiliations:** School of Chinese as a Second Language, Peking University, Beijing, China; Centre for Mental Health & Brain Sciences, Swinburne University of Technology, Hawthorn, VIC, Australia; Centre for Cognitive and Brain Sciences, University of Macau, Macau SAR, China; Changping Laboratory, Beijing, China; Center for MRI Research, Academy for Advanced Interdisciplinary Studies, Peking University, Beijing, China; Laboratory of Medical Physics and Engineering, School of Physics, Peking University, Beijing, China; McGovern Institute of Brain Research, Peking University, Beijing, China; National Biomedical Imaging Center, Peking University, Beijing, China

**Keywords:** optically pumped magnetometers, magnetoencephalography, epilepsy, clinical translation, naturalistic neuroscience, wearable neuroimaging

## Abstract

Optically pumped magnetometer magnetoencephalography (OPM-MEG) has moved beyond proof of principle. The decisive question is no longer whether cryogen-free sensors can detect neuromagnetic fields from the human brain with sufficient sensitivity, but whether complete systems can deliver clinical decision value and interpretable recordings during more naturalistic behaviour. Here we argue that OPM-MEG is developing along two partly overlapping trajectories. One is clinical translation, where usefulness depends more on reliable workflows, calibrated source localisation, auditable quality control, and empirical evidence for improved diagnostic or treatment decisions. The other is naturalistic neuroscience, where wearable measurement can connect neural dynamics to movement, action, and social behaviour without conflating neural activity with movement-related artefacts. Both trajectories depend on a shared engineering stack including magnetic field control, dense spatial sampling, accurate calibration, integrated electronics, artefact suppression, participant comfort, and synchronised behavioural measurement. The next stage of OPM-MEG should be evaluated by clinical utility in specific patient groups, statistically identifiable neural dynamics in behaviourally rich settings, and sufficient system detail for replication across systems and sites.

## 1 Introduction

Magnetoencephalography (MEG) provides a non-invasive, millisecond-resolution measure of human electrophysiology by recording magnetic fields generated by neuronal currents (Baillet, 2017; Hämäläinen et al., 1993). Conventional systems use superconducting quantum interference devices (SQUIDs), which reach very low noise floors of roughly 2–4 fT/√Hz but require cryogenic cooling to about -269°C. Because the sensors must remain inside a cryogenic dewar, the sensor array is fixed and separated from the scalp by several centimetres of thermal insulation. Although paediatric and infant SQUID systems exist, each helmet remains a single-size rigid shell, and most installations offer only an adult-sized helmet. SQUID-MEG has enabled decades of productive neuroscience and clinical studies, while constraining the populations, anatomies and behaviours that can be measured (Brookes et al., 2022). The reliance on liquid helium, heavy magnetically shielded rooms, and dedicated site infrastructure also makes SQUID-MEG expensive to purchase and operate, which has confined the technique to a small number of sites worldwide (Baillet, 2017).

Optically pumped magnetometers (OPMs) relax several of these constraints. Most OPM-MEG systems use an alkali vapour (e.g., ^87^Rb) heated to about 150°C and operate in the spin-exchange relaxation-free (SERF) regime (Kominis et al., 2003). They require no cryogenics, reach noise floors near 10 fT/√Hz, and can be placed within millimetres of the scalp, where neuromagnetic fields are larger in amplitude and more spatially focal than SQUID-MEG (Boto et al., 2016; Iivanainen et al., 2017; Tierney et al., 2019). Unlike a SQUID array, each OPM is a compact self-contained sensor that can be positioned independently, so sensors can be arranged in rigid or soft helmets, and the array can be customised to individual head sizes and regions of interest. Because they are small and wearable, OPM arrays can in principle move with the participant when residual magnetic fields are adequately controlled (Boto et al., 2018). Moreover, OPM systems do not consume liquid helium and can operate with lighter shielding, they also promise lower purchase and running costs, which could widen access to MEG (Brookes et al., 2022; Schofield et al., 2024). Earlier demonstrations emphasised signal detectability, proximity to cortex, and movement tolerance. The field has now entered a deployment phase in which sensor performance is only one component of value. Magnetic field control, sensor array design, calibration, system integration, analysis workflow, and participant comfort determine whether OPM-MEG effectively solves a specific clinical or scientific problem.

The relevant question is, therefore, whether an OPM-MEG system can solve a specific research problem better than the alternatives available for that use case. An accessible alternative to OPM-MEG is high-density electroencephalography (HD-EEG), which is also wearable and records at the same millisecond resolution (Michel & Brunet, 2019). Magnetic permeability is essentially uniform across brain, skull, scalp, and the air between head and sensors, whereas electrical conductivity differs sharply between these tissues and cannot be measured in vivo. MEG topographies are, therefore, less smeared than EEG potentials, and MEG source estimates are less biased by head-model approximations, despite the reduced sensitivity to radially oriented currents (Baillet, 2017; Hämäläinen et al., 1993). OPM-MEG retains that advantage in a wearable form, though with a higher cost than HD-EEG and a requirement for magnetic shielding, so the choice between them should be argued explicitly for each application.

These trade-offs motivate an application-specific framework for evaluating OPM-MEG, centred on where it offers distinctive value and what is required to realise that value in practice. This Perspective develops that framework through three linked claims. First, OPM-MEG should be evaluated by use case rather than by a general comparison against SQUID-MEG. Second, the field is developing along two especially important and partly overlapping trajectories: clinical translation and naturalistic neuroscience. Third, both trajectories depend on the same system-level foundations. The shared bottlenecks are no longer simply whether a single OPM sensor is sensitive enough, but whether the full instrument supports reproducible measurement, efficient setup, auditable quality control, and interpretable results across participants, tasks, and sites.

A useful conceptual model places OPM-MEG in a two-dimensional development space (Fig. 1). One axis is translational maturity, or how close a system is to routine and accountable use, running through proof of principle, technical and analytical validity, application-specific validity, workflow integration, and clinical decision impact or scientific inference value. The other axis is behavioural freedom, or how much a participant can move and behave naturally while brain activity is recorded, running from constrained seated tasks to head movement, whole-body movement, multi-subject interaction, and open-space behaviour. This framing preserves the context supplied by SQUID-MEG. Conventional SQUID-MEG remains a mature technology with established workflows for clinical applications and neuroscience research, very low sensor noise, robust analysis pipelines, and decades of methodological development. OPM-MEG offers a different design space. Its advantages become most compelling when the question benefits from on-scalp measurement, flexible placement, movement tolerance, potentially lower infrastructure burden, or deployment outside a conventional MEG system.

**Fig. 1.**
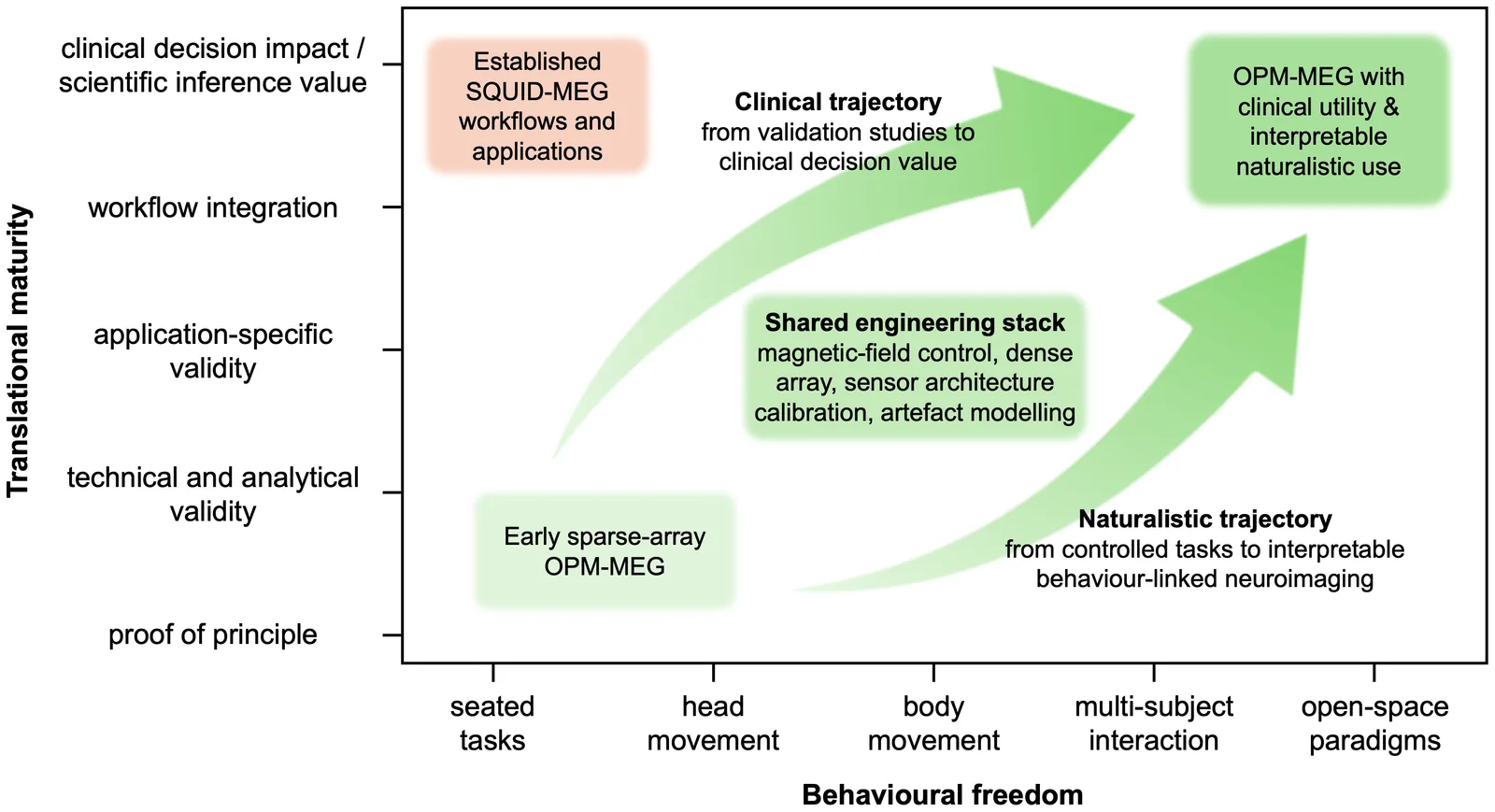
Conceptual map of the two development trajectories in OPM-MEG. The vertical axis represents translational maturity, progressing from proof of principle through technical and analytical validity, application-specific validity, and workflow integration to clinical decision impact or scientific inference value. These milestones apply to both clinical and neuroscience applications. The horizontal axis represents increasing behavioural freedom, from seated tasks to open-space settings. Earlier sparse-array OPM-MEG systems established proof of principle, while established SQUID-MEG workflows and applications, shown in orange, mark a mature reference point. Starting from the earlier sparse-array OPM-MEG, the technique is advancing along two partly overlapping trajectories. The clinical trajectory moves from concordance studies towards clinical decision value, and the naturalistic trajectory moves from controlled tasks towards interpretable behaviour-linked neuroimaging. Both trajectories depend on a shared engineering stack and rise through the same milestone levels towards the target region of mature OPM-MEG with clinical utility and interpretable naturalistic use. Note that positions and trajectories in this conceptual map are schematic and conceptual rather than quantitative.

Both clinical translation and naturalistic neuroscience trajectories originate from the earlier sparse-array OPM-MEG and rise through these milestone levels towards the goal of mature OPM-MEG with clinical utility and interpretable naturalistic use. Progress along the behavioural-freedom axis does not bypass the milestones of the maturity axis. Each new application, whether clinical or naturalistic, must itself move through proof of principle, technical validity, and validation before routine use, tracing its own pathway through the same milestones. The top milestone is reached when a measurement changes a clinical decision or supports a scientific conclusion that could not otherwise be drawn.

## 2 Clinical Translation: From Detectability to Decision Value

Clinical translation begins with signal detection and matures through utility. A clinically useful OPM-MEG system operates predictably in hospital settings, accommodates variable anatomy and clinical states (e.g., resting-state, tasks that require movement, and clinical seizures during the recording), completes preparation and acquisition within realistic time constraints, and generates outputs that clinicians can interpret within an established decision framework. This decision framework is the existing clinical pathway into which the examination fits, such as the multidisciplinary presurgical evaluation of epilepsy, where MEG findings are weighed alongside seizure semiology, MRI, and EEG to plan stereo-EEG implantation or resective surgery. Moreover, a clinically robust system must be operable by hospital technologists and staff with shared responsibilities rather than only by OPM specialists, which requires standardised acquisition protocols, automated quality checks, and predictable failure modes. In clinical populations, artefacts from prior implants, neurostimulators, and other hardware often cannot be avoided, therefore, acquisition and analysis pipelines must remain usable in their presence (Hnazaee et al., 2023). The comparison with established SQUID-MEG should focus on workflow-level added value such as greater patient compatibility, more targeted anatomical coverage, improved recording feasibility, or clearer actionability for a specific clinical application.

Epilepsy is the strongest near-term proving ground because conventional SQUID-MEG already has an established role in the non-invasive presurgical evaluation of medically intractable localisation-related epilepsy, with clinical practice standards for recording, analysis, and reporting (Bagić et al., 2011a, 2011b; Burgess et al., 2011). On-scalp OPM-MEG extends this logic to patients and source configurations that are challenging for fixed SQUID helmets, including small paediatric heads that sit far from fixed sensors, anterior and mesial temporal or orbitofrontal foci that lie near or beyond the rim of a standard helmet, and deep sources whose fields decay steeply with distance. Paediatric recordings show diagnostic feasibility in school-aged children with improved fit and larger measured signal amplitudes, although earlier studies also had limited sensor number and coverage (Feys et al., 2022). Ictal and interictal recordings show access to spontaneous epileptiform activity rather than only evoked-response benchmarks (Hillebrand et al., 2023). Temporal-lobe studies demonstrate that combined scalp and face-mounted OPMs can detect and localise temporal interictal epileptiform discharges, with face-mounted sensors improving coverage of anterior and mesial temporal regions (Feys et al., 2025). Mesial temporal generators have also been evidenced by OPMs recorded simultaneously with stereo-EEG, which captured interictal discharges arising from hippocampal and amygdala structures (Schwartz et al., 2025). Deep generators can be detected by MEG under favourable conditions, but their detection and localisation depend strongly on sensor proximity and noise (Bénar et al., 2021). Recent comparative studies keep pushing the field beyond isolated demonstrations. OPM-MEG recordings have also produced interictal-discharge source estimates consistent with SQUID-MEG and have enabled comparison with stereo-EEG contacts or surgical and thermocoagulation targets (Chen et al., 2025).

These studies strengthen the case that OPM-MEG can reproduce the information on which current clinical practice relies, in particular the detection of interictal epileptiform discharges and the localisation of the irritative zone used, together with other modalities, to plan stereo-EEG implantation and resective surgery. Such concordance with SQUID-MEG and intracranial recordings is nevertheless not the same as clinical decision value. The decision value of SQUID-MEG in presurgical evaluation is supported by practice guidelines and outcome studies, whereas for OPM-MEG it remains to be shown that the measurements change diagnostic conclusions or treatment plans rather than merely agree with existing modalities.

OPM-MEG systems based on helium add useful breadth to the clinical design space. In patients already undergoing intracranial stereo-EEG monitoring, simultaneous helium OPM recordings showed that epileptic abnormalities visible on stereo-EEG could also be detected by the extracranial OPM sensors. Most interictal discharges seen with SQUID-MEG have also been detected with helium OPMs in the same patients, with comparable signal-to-noise ratio and similar spike morphology (Schwartz et al., 2025). In addition, a case comparison of triaxial rubidium and helium OPMs found that most interictal discharges were detectable with both sensor types (Badier et al., 2023; Feys et al., 2023). Although these studies are not yet proof of broad clinical utility, they expand the plausible sensor choices for patient-compatible epilepsy workflows that patients can actually complete, with helmets that fit children or accommodate implanted devices, tolerance of movement during seizures, and recording durations compatible with clinical schedules. They also make clear that OPM-MEG is not one monolithic technology.

The strongest clinical cases will probably be those in which OPM-MEG changes the feasibility or interpretation of the examination, not merely the detected amplitude of a spike. Examples include children who fit poorly in a fixed adult helmet, patients whose seizures or movement disorder make immobility unrealistic, and temporal or orbitofrontal foci that benefit from targeted face or inferior-array coverage. For these cases, the relevant comparator is not an ideal SQUID recording from a still, cooperative adult, but the recording that can realistically be delivered in the clinic, which may be shortened, degraded by movement, acquired under sedation, and in some patients cannot be obtained at all. Against that benchmark, a wearable system that returns an interpretable recording where the conventional SQUID-MEG fails is already an advance.

The next-level evaluation for OPM-MEG’s clinical application is prospective decision value. Evidence for decision value can take several concrete forms, including a change in diagnostic confidence, a revised stereo-EEG implantation strategy or surgical hypothesis, altered resection or ablation planning, shorter monitoring duration, fewer inconclusive examinations, improved patient tolerability, or reduced cost. Because SQUID-MEG has already demonstrated influence on several of these decisions, a decisive OPM-MEG study must show additional value beyond it, for example, by recording ictal activity as wearable arrays tolerate movement during seizures, by improving coverage of mesial temporal or orbitofrontal generators through targeted sensor placement, or by delivering comparable information at lower cost and infrastructure burden.

Similar caution is needed outside epilepsy. Movement disorders, dementias, schizophrenia-spectrum disorders, autism, and attention-deficit/hyperactivity disorder all involve abnormal neural dynamics, and all affect patient groups for whom fixed helmets, long immobile sessions, and scarce scanner access are practical barriers to conventional MEG. Thus, wearable OPM-MEG is a natural candidate across this range (Brickwedde et al., 2024). The common rationale is patient compatibility rather than disease specificity, and each condition remains subject to the same evidential standard. Moreover, exploratory biomarker findings should not be presented as clinical utility before decision-relevant endpoints have been tested.

## 3 Naturalistic Neuroscience: From Richer Stimuli to Richer Behaviour

The second trajectory focuses on the experimental paradigm scope of human neuroimaging. Many cognitive neuroscience studies require participants to sit still, view a screen, and respond through button presses. That abstraction remains powerful for controlled inference, but it captures only a limited portion of human behaviour (Stangl et al., 2023). Spatial navigation, active sensing, posture, social interaction, gait, motor learning, and daily communication involve continuous coupling among brain, body, and environment. Wearable OPM-MEG provides a route for studying these processes with spatiotemporally resolved neural activity and more intact behavioural structure.

The key shift is from richer stimuli to richer behaviour. In earlier landmark work, wearable OPM-MEG recorded interpretable sensorimotor responses while participants nodded, stretched, drank, and played a ball game inside a shielding room, and later studies recorded neural activity from standing, mobile participants and systematically characterised the interference-suppression techniques that such recordings require (Boto et al., 2018; Seymour et al., 2021, 2022). Virtual-reality integration extends the method towards navigation and active visual exploration, from an earlier proof of concept with projected visual stimulation to a recent integrated platform that synchronises immersive virtual environments with wearable recordings (Roberts et al., 2019; Zabbah et al., 2026). Combined video telemetry and wearable MEG, with pose estimation and deep-learning-based behavioural annotation, now allows events to be defined by body movement as well as by stimulus timing (O’Neill et al., 2025). In this framework, a neuroimaging event can be a reach, a postural transition, a locomotor phase, a gaze shift, or even an interactional turn.

Naturalistic OPM-MEG is, therefore, behaviour-linked neuroimaging. Video, motion capture, eye and head-position tracking, electromyography, task-state annotation, and OPM-MEG together provide a multimodal description of perception, action, and cognition as they unfold in time. The same logic extends to higher-level cognition where decoding and representational analyses can ask how categorical information, memory, social cues, and action plans evolve during behaviour rather than only after externally timed stimuli. This is where OPM-MEG offers a distinct contribution to human cognitive neuroscience, because the temporal structure of neural activity can be related to the temporal structure of self-generated behaviour (O’Neill et al., 2025; Stangl et al., 2023). Naturalistic experimental designs complement controlled experiments rather than replacing them. It is now feasible to combine constrained paradigms that establish mechanism with richer paradigms that test whether the same neural dynamics can be observed during active behaviour (Boto et al., 2018; Seymour et al., 2021). OPM-MEG is well suited to this iterative strategy because the same participant can be measured in seated, head-moving, and more embodied task variants, allowing experimental control and ecological structure to be varied systematically.

Validation for this naturalistic trajectory deserves particular attention for behaviours that lack conventional neuroimaging comparators. Practical strategies include benchmarking against HD-EEG under the same paradigm (Michel & Brunet, 2019), anchoring new paradigms to constrained task analogues with established effects, demonstrating within-subject replication across repeated sessions, and, where relevant, comparing with invasive recordings in patients. Concordance between on-scalp OPM recordings and simultaneous intracranial stereo-EEG in epilepsy patients illustrates how invasive recordings can help validate the neural origin and interpretation of wearable OPM signals (Badier et al., 2023; Schwartz et al., 2025).

Movement compatibility also creates several issues. Once participants move, neural activity covaries with factors related to the task as well as the residual magnetic fields. Considerable progress has been made on the instrumental side of this issue. Bi-planar and matrix-coil systems null the background field around the head and can compensate movement-induced field changes with low latency during ambulatory recordings (Holmes et al., 2023; Rea et al., 2021). Post-acquisition methods provide a complementary layer of correction. Homogeneous field correction suppresses spatially homogeneous interference, whereas adaptive multipole modelling explicitly models the spatial patterns associated with brain signals and environmental interference to support their separation and improve signal-to-noise ratio (Tierney et al., 2021, 2024).

This separation of brain-generated fields from external magnetic interference does not, however, resolve a distinct identifiability problem. Neural effects of interest may still covary with head and body motion, task variables, ocular and muscular activity, and other physiological signals. Addressing this problem requires explicit nuisance models, synchronised behavioural measurement, and statistical designs that separate correlated predictors. Transparent quality control is equally important and should report residual field values, sensor dropouts, calibration status, head-motion summaries, and artefact rates with the neural data. In practice, nuisance models may include pose, head-position, eye, and muscle regressors together with field-control logs. These models should be supported by clear event definitions, non-neural reference channels where available, cross-session replication, and source-space validation during movement. Ecological richness is scientifically informative only when the neural interpretation remains identifiable (Seymour et al., 2022).

Brain–computer interfaces (BCIs) form a bridge between naturalistic neuroscience and clinical translation because a BCI decodes neural signals associated with self-generated intentions and actions in real time. Movement-compatible acquisition, artefact control, and synchronised behavioural measurement, therefore, shared methodological requirements that can help translate naturalistic research into clinically useful interfaces. OPM-MEG studies have demonstrated real-time neural interfacing and single-trial decoding, including spelling paradigms and high-frequency steady-state visual evoked fields (Ji et al., 2024; Wittevrongel et al., 2021). These proof-of-concept studies are not yet evidence for routine deployment. Near-term opportunities are, therefore, likely to emerge first in constrained settings where reliable decisions have substantial value despite limited communication bandwidth. Potential applications include visual target selection, communication support for people with motor impairments, rehabilitation feedback, and the assessment of closed-loop neuromodulation. Hybrid interfaces that integrate OPM-MEG with EEG, eye tracking, or electromyography may further improve robustness and practical usability.

## 4 The Shared Engineering Stack

The ultimate goals of clinical translation and naturalistic neuroscience differ, but their underlying engineering bases are largely shared (Table 1). This section organises these shared requirements according to how directly they constrain current OPM-MEG systems. Magnetic field control is the first because all subsequent capabilities depend on sensors remaining within their dynamic range in the intended recording environment. Dense spatial sampling and array-level validation come next as they determine the spatial information available for further analysis. Sensor architecture and calibration follow because sensor design determines the achievable bandwidth, dynamic range, and ergonomics. Calibration converts a flexible dense array into a quantitative measurement system. System integration, including synchronised behavioural measurement and video recording, completes the stack by turning laboratory setups into deployable platforms. Artefact suppression and behavioural modelling, discussed in Section 3, are essential for converting movement-compatible recordings into interpretable neural results. Table 1 summarises these requirements in the same sequence and ranks them by near-term priority.

**Table 1.** Shared system requirements for the two trajectories of OPM-MEG.

	Clinical translation	Naturalistic neuroscience	Bottlenecks
Magnetic field control	Predictable sensor operation in magnetically noisy hospital environments	Acquisition that remains stable while participants move through residual fields	Residual static fields, field gradients, and ambient interference
Dense spatial sampling	Accurate localisation of abnormal brain function, whole-head coverage, and fit to individual anatomy	Separation of neural sources from artefacts during movement and behaviour	Sensor cost, per-sensor calibration burden, inter-sensor crosstalk, and coverage of the head surface
Sensor architecture	High sensitivity, manageable scalp heat load, and arrays targeted to clinical regions of interest	Greater bandwidth, larger dynamic range, participant comfort, and wearable ergonomics	Matching sensor type to task and workflow
Calibration	Auditable and reproducible source reconstruction for clinical reporting	Comparable measurements across sessions despite flexible sensor placement	Uncertainty in sensor geometry, gain, and sensitive-axis orientation
Artefact and behaviour modelling	Reproducible and transparent data quality control	Identifiable neural inference during self-generated movement	Nuisance modelling and synchronisation of multimodal behavioural data

Magnetic field control remains the most stringent constraint on OPM-MEG deployment. OPMs are highly sensitive to residual magnetic fields and gradients, environmental interference, nearby electronics, and movement within incompletely compensated fields. Matrix-coil active shielding can combine optical tracking with low-latency compensation for motion-related field changes, supporting source localisation during translations and rotations (Holmes et al., 2023). Lightly shielded rooms, when combined with active field control and signal-separation processing, can provide data suitable for evoked-response and source-level analyses (Holmes et al., 2025). Such systems require less demanding infrastructure than high-performance magnetically shielded rooms. Recordings of cortical activity in unshielded or lightly shielded environments indicate a longer-term pathway towards further reductions in shielding requirements (Limes et al., 2020; Zhang et al., 2020). Two recent studies mark rapid progress towards this goal. A compact and lightweight system combined active shielding with software denoising to record brain activity without a magnetically shielded room (Bezsudnova et al., 2026), and a wearable system using room-scale active coils and on-scalp gradiometers localised sensory evoked responses and tracked motor-cortex activity during smartphone use in an ordinary office (Zheng et al., 2026). Despite these advances, whole-head recordings over extended periods in unshielded daily environments remain an ambitious goal rather than a routine capability.

High-density sampling is central because on-scalp sensors measure neuromagnetic fields much closer to their sources, where the spatial frequency content of the field pattern is higher. This effect is most pronounced for superficial neocortical sources on the convexity and weaker for deep sources, and it means that dense arrays are needed to capture the additional spatial detail that proximity makes available (Iivanainen et al., 2017). An integrated 128-sensor OPM-MEG system demonstrates full-head coverage, sensor-performance characterisation, and cross-validation with established paradigms (Alem et al., 2023). Besides, an 80-sensor system has produced evoked source-level responses, resting-state power distributions, functional connectomes, and microstate patterns aligned with established MEG measures (Xu et al., 2025). More recently, a 384-channel triaxial system further illustrates how channel count, synchronised electronics, and rapid array calibration can improve spatial sampling, sensitivity, and cortical coverage (Schofield et al., 2025).

System validation is also moving from illustrative examples towards broader characterisation. Large-sample, multi-task comparisons have shown similar evoked responses, field patterns, and oscillatory dynamics between OPM-MEG and SQUID-MEG across healthy participants and individuals with neurological disorders (Wang et al., 2024). Such work is important because OPM-MEG gains credibility through reproducible behaviour across tasks and cohorts alongside single-study demonstrations. Validation across participants, sites, and analysis pipelines will become especially valuable as arrays grow denser and claims become more specific.

OPM-MEG now comprises different system architectures. Most wearable systems use alkali vapour sensors based on rubidium, although caesium-based devices have also been developed, which combine high sensitivity with increasingly compact sensor heads. These sensors are cryogen free but not literally unheated. Alkali vapour cells require heating and thermal management. Helium-based OPMs occupy a smaller but relevant part of the OPM sensor design space. They provide room-temperature operation without vapour cell heating, native vector measurement, broad bandwidth, and large dynamic range, with demonstrations spanning proof-of-concept systems, healthy volunteer comparisons, epilepsy recordings, and whole-head performance assessment (Bonnet et al., 2025; Fourcault et al., 2021; Gutteling et al., 2023; Labyt et al., 2019). Their current limitations include relatively lower sensitivity, less miniaturised hardware, and a smaller evidence base than alkali vapour systems. In practice, sensor choice affects heat load, bandwidth, dynamic range, axis geometry, sensor-to-scalp distance, helmet ergonomics, magnetic field operating regime, and calibration, all of which interact with the clinical and neuroscience applications.

Calibration is another decisive factor. Unlike the fixed geometry of a SQUID helmet, OPM sensor arrangement can vary across helmets, participants, and sessions. Sensitive axes and gains can deviate from nominal values, and ambient magnetic fields can alter effective response properties. Recent calibration methods based on well-characterised magnetic fields estimate sensor geometry, orientation, and gain with millimetre-level and degree-level agreement between independent approaches, improving beamformer signal-to-noise ratio relative to assumed coordinates (Hill et al., 2025). Thus, accurate calibration is essential for dense arrays, flexible caps, source localisation, and movement-compatible experiments.

System integration determines whether an OPM-MEG instrument functions as a deployable platform. Portable electronics, synchronised acquisition, cable and heat management, optical scanning, motion capture, video recording, and acquisition software shape setup efficiency, participant comfort, and cross-site reproducibility. Synchronised video deserves emphasis for clinical use because it allows the clinical manifestations of a recording to be assessed relative to the traces, such as seizure semiology relative to epileptiform activity. Acquisition software should also support real-time filtering, visualisation, and review during data collection. These functions improve patient safety by helping operators identify events such as prolonged subclinical seizures and initiate ictal testing when clinically indicated. Portable integrated electronics already support more robust and transportable data acquisition (Schofield et al., 2024). Wireless OPM-MEG has demonstrated both spontaneous and evoked responses using non-magnetic electronics and tailored communication protocols (Cheng et al., 2024). Scaling such designs to dense arrays needs further efforts on synchronisation, bandwidth, electromagnetic compatibility, and power management.

## 5 Milestones and Bottlenecks

The dual-trajectory framing separates common OPM-MEG system development from application-specific goals. Clinical applications emphasise patient compatibility, standardised reporting, localisation accuracy, workflow integration, and measurable consequences for diagnosis or treatment planning. Naturalistic neuroscience applications depend on mobility, behavioural measurement, multimodal synchronisation, and models that retain the temporal structure of action while preserving reliability and interpretability. Progress towards clinical translation requires prospective studies that measure decision value against predefined endpoints, demonstrations of artefact robustness in patients with implants, feasibility studies of research workflows in routine hospital settings, and reporting of implementation and operating cost. For naturalistic neuroscience, the priorities are validation of new paradigms against constrained analogues and independent modalities, automated behavioural annotation, explicit nuisance modelling, and auditable quality control standards across studies and sites. Considering these trajectories together reveals their shared technical foundation, while keeping them distinct prevents evidence from one context from being overextended to another. For example, movement-compatible recording does not by itself establish clinical maturity, and accurate clinical localisation does not by itself establish readiness for complex behavioural environments.

The principal failure modes are also conceivable. Clinical OPM-MEG could stall if studies demonstrate concordance with SQUID-MEG or intracranial recordings without showing added decision value. Naturalistic OPM-MEG could produce visually compelling experiments whose neural interpretations remain confounded by movement and uncontrolled behavioural covariance. Dense arrays could become impressive but difficult to calibrate, maintain, or reproduce. Biomarker studies could overstate diagnostic value before multisite validation and outcome linkage. Therefore, a persuasive study should pair technical foundation with a clearly stated endpoint. In clinical applications, the endpoint may be a changed surgical hypothesis, a better tolerated paediatric examination, or a reduction in inconclusive recordings. In naturalistic neuroscience research, the endpoint may be a model that predicts neural activity from measured behaviour while controlling for artefacts and correlated task variables. In engineering-related work, the endpoint may be setup time, calibration accuracy, or real-time magnetic field control performance. These endpoints keep OPM-MEG systems anchored to deployment rather than demonstration alone.

## 6 Conclusion

The future of OPM-MEG will be decided by whether complete systems can gain more insights along the two promising trajectories: decision-relevant clinical workflows and interpretable recordings during more naturalistic behaviour. The clinical trajectory demands reliability, standardisation, and demonstrated utility in various patient groups. The naturalistic trajectory demands movement-compatible measurement combined with rigorous behavioural modelling and explicit nuisance control. Advances in dense sampling, calibration, magnetic field control, artefact suppression, participant comfort, and system integration are needed to support both trajectories. As these capabilities converge, OPM-MEG can broaden MEG from a fixed scanner for constrained electrophysiology into an adaptable platform for clinical decision making and human behaviour in action.

## Data Availability

No code or data are associated with this work.
